# Brief Maternal Separation Inoculates Against the Effects of Social Stress on Depression-Like Behavior and Cocaine Reward in Mice

**DOI:** 10.3389/fphar.2022.825522

**Published:** 2022-03-11

**Authors:** C. Calpe-López, M. A. Martínez-Caballero, M. P. García-Pardo, M. A. Aguilar

**Affiliations:** ^1^ Neurobehavioural Mechanisms and Endophenotypes of Addictive Behaviour Research Unit, Department of Psychobiology, University of Valencia, Valencia, Spain; ^2^ Department of Psychology and Sociology, Faculty of Social Sciences, University of Zaragoza, Teruel, Spain

**Keywords:** anxiety-like behaviour, cocaine, conditioned place preference, depression-like behaviour, maternal separation, mice, social defeat, stress inoculation

## Abstract

Exposure to intermittent repeated social defeat (IRSD) increases the vulnerability of mice to the rewarding effects of cocaine in the conditioned place preference (CPP) paradigm. According to the “inoculation of stress” hypothesis, a brief period of maternal separation (MS) can provide protection against the negative effects of IRSD. The aim of the present study was to assess whether exposure to a brief episode of MS prevents the subsequent short-term effects of IRSD on depression- and anxiety-like behaviors and to explore its long-term effects on cocaine CPP in mice. Four groups of male C57BL/6 mice were employed; two groups were separated from their mother [6 h on postnatal day (PND) 9], while the other two groups were not (controls). On PND 47, 50, 53 and 56, mice that had experienced MS were exposed to social defeat in the cage of an aggressive resident mouse (MS + IRSD group) or were allowed to explore an empty cage (MS + EXPL group). The same procedure was performed with control mice that had not experienced MS (CONTROL + IRSD and CONTROL + EXPL groups). On PND57-58, all the mice performed the elevated plus maze and the hole-board, social interaction and splash tests. Three weeks after the last episode of defeat, all the mice underwent the CPP procedure with cocaine (1 mg/kg). Irrespective of whether or not MS had taken place, a reduction in open arms measures, dips, and social interaction was observed in mice that experienced IRSD. A higher latency of grooming and acquisition of cocaine-induced CPP were observed only in mice exposed to IRSD alone (CONTROL + IRSD). These results suggest that exposure to a brief episode of stress early in life increases the subsequent resilience of animals to the effects of social stress on vulnerability to cocaine.

## 1 Introduction

In spite of cumulative evidence of the potential risks of drug abuse, cocaine is widely consumed among adolescents and young adults (European Monitoring Centre for Drugs and Drug Addiction, 2020). It is clear that biological factors can predispose an individual to cocaine addiction; however, different animal models have demonstrated that environmental factors are also involved (Badiani and Spagnolo, 2013; El Rawas and Saria, 2016; Montagud-Romero et al., 2018; Ahmed et al., 2020).

Among these environmental factors, stress - understood as adversity/negative experiences in life—has been shown to enhance vulnerability to the rewarding effects of cocaine and other drugs of abuse (Aguilar et al., 2013; Rodríguez-Arias et al., 2013; Vannan et al., 2018). Among the different types of stress, social stress is currently the most common, and can be modelled in experimental animals with the chronic/repeated social defeat (SD) paradigm, which is known to have predictive power (Vasconcelos et al., 2015; Patel et al., 2019; Wang et al., 2021; García-Pardo et al., 2022). Several studies have shown an increase in the rewarding effects of cocaine in the conditioned place preference (García-Pardo et al., 2019; Calpe-López et al., 2020; Montagud-Romero et al., 2020) and self-administration (Holly EN. et al., 2016; Rodríguez-Arias et al., 2017) paradigms among animals exposed to SD. In a recent study, we observed that mice exposed to SD displayed anxiety- and depression-like behaviours, social avoidance and greater stress reactivity (Calpe-López et al., 2020).

Another intervention that induces social stress is interference with the maternal-offspring relationship, which has an essential influence on the development of mammals. After birth, pups are vulnerable and the mother carried out important functions, such as protection, warming and feeding, in order to guarantee the physical health and survival of their offspring (Numan and Insel, 2003). Several research works have demonstrated that inadequate maternal care has devastating consequences for the maturation of the central nervous system and mental health of the pups (Yang et al., 2017; Courtiol et al., 2018). In this sense, maternal separation (MS) stress constitutes a critical experience that can induce behavioural alterations and neuropsychiatric disorders in later life (George et al., 2010; Gracia-Rubio et al., 2016; Lukkes et al., 2017; Zhou et al., 2020). Repeated episodes of MS (4–8 h per day, from postnatal day (PND) two to PND16) have been shown to increase the vulnerability of offspring to the rewarding effects of drugs of abuse during adolescence or adulthood (Delavari et al., 2016; Viola et al., 2016; Orso et al., 2017; Castro-Zavala et al., 2020; Castro-Zavala et al., 2021a; Castro-Zavala et al., 2021b; Arenas et al., 2022). Furthermore, different procedures of repeated MS (for 4–8 h per day, from PND2 until PND12-20) enhance the anxiety of adolescent mice in the elevated plus maze (EPM) (Shin et al., 2016; Wang et al., 2017) and in the social preference test (Wang et al., 2017) and induce anhedonia in the saccharin preference test (Wang et al., 2017) and depression-like behaviour in the forced swimming test (He et al., 2020). Moreover, exposure to MS (3 h/day, PND 1–14), though it did not alter the behaviour of mice by itself, was seen to increase the risk of depression-like behaviours in the forced swimming and sucrose preference tests when mice were exposed to an additional restraint stress in late adolescence (PND 42–56) (Han et al., 2019).

However, stress is not necessarily negative, as it can have adaptive properties and induce responses aimed to improve the physical and psychological functioning of the individual (Southwick and Charney, 2012; Brockhurst et al., 2015). In early life, while not of an excessive magnitude, a stressful episode can promote resilience to subsequent stressful experiences later in life (Lyons et al., 2010; Daskalakis et al., 2013; Ashokan et al., 2016). In fact, although resilience is an innate capacity, it is not a stable trait, but rather is a dynamic process that develops throughout a life span (Rutter, 2012; Kalisch et al., 2019) and can be enhanced by different factors (Calpe-López et al., 2022, in press). This could explain why some people rebound after adverse situations while others develop a mental disorder and never recover (Charney, 2004; Yao and Hsieh, 2019). In this sense, exposure to mild or moderate stressors can induce an adaptive stress response in the individual, increasing his/her resilience to the negative effects of future stressful events (Russo et al., 2012; Southwick and Charney, 2012).

Repeated SD has proven itself to be a useful animal model for studying resilience to the negative consequences of social stress and the mechanisms which are involved (Krishnan et al., 2007; Hodes et al., 2014; Henry et al., 2018). A recent study carried out in our laboratory demonstrated that some mice are resilient to the effects of intermittent repeated SD (IRSD) on cocaine reward (Calpe-López et al., 2020). We observed that exposure to IRSD increased the rewarding effects of cocaine in the CPP paradigm, but mice with certain behavioural traits showed resilience to the negative effects of stress (Calpe-López et al., 2020). However, how exposure to an episode of stress in early life affects the subsequent effects of social stress on cocaine reward in later life has not been studied. Thus, the objective of this work was to determine if a brief MS in early life modifies the behavioural response to IRSD in late adolescence and can reverse the potentiating effects of social stress on the rewarding effects of cocaine in adulthood. For this purpose, experimental groups were exposed to MS, IRSD, MS + IRSD or did not undergo stress. Different behavioural tests (EPM, hole-board, social interaction, and splash tests) were employed in order to determine the behavioural effects of both types of stress exposure in late adolescent animals. Three weeks after the last episode of defeat, acquisition of CPP following conditioning with cocaine was evaluated in all the groups. Our hypothesis was that a brief MS in early life can inoculate against the negative effects of subsequent stress and promote resilience to anxiety-, depression- and addiction like symptoms induced by IRSD.

## 2 Material and Methods

### 2.1 Animals

Forty-nine male mice of the C57BL/6 strain (born in the Psychology Department Laboratory, University of Valencia, from parents acquired from Charles River, France) and 15 male mice of the OF1 strain (Charles River, France) were used in the present study. They were housed by litter with mother and siblings in plastic cages (25 cm × 25 cm × 14.5 cm). Later, they were weaned and separated from the female mice on PND 21, but remained grouped by litter (3–6 male mice). Mice used as aggressive opponents (OF1) arrived in the laboratory on postnatal day (PND) 21 and were housed individually in plastic cages (23 cm × 32 cm × 20 cm) for three or more weeks before initiation of the experimental procedures in order to induce heightened aggression (Rodríguez-Arias et al., 1998). All mice lived under constant temperature (21°C), a reversed 12 h light schedule (on 19:30–07:30) and food and water ad libitum. Before initiation of social defeat or exploration, experimental mice were handled (5 min/day, for 3 days) in order to decrease their stress response to manipulation. All protocols were conducted in compliance with Directive 2010/63/EU and were approved by the Ethics Committee in Experimental Research (Experimentation and Animal Welfare) of the University of Valencia (A1507028485045).

### 2.2 Drugs

For place conditioning, mice received intraperitoneal injections (0.01 ml/g of body weight) of cocaine (Alcaliber Laboratory, Madrid, Spain) or physiological saline (NaCl 0.9%) (the same as that used to dissolve the drug). On the basis of previous studies, we employed a dose of 1 mg/kg of cocaine (García- Pardo et al., 2019; Calpe-López et al., 2020).

### 2.3 Experimental Design

Experimental mice (C57BL/6) were assigned to four groups according to the type of stress experienced in early-life (PND 9) and late adolescence (PND 47, 50, 53 and 56). The first group was exposed to a brief maternal separation and subsequently to four episodes of social defeat (MS + IRSD, *n* = 21); the second group was exposed to MS but did not experience social stress during adolescence (MS + EXPL, *n* = 12); the third group was not exposed to MS but experienced IRSD in late adolescence (CONTROL + IRSD, *n* = 8); and the fourth group was not exposed to MS or IRSD (CONTROL + EXPL, *n* = 8).

**FIGURE 1:**
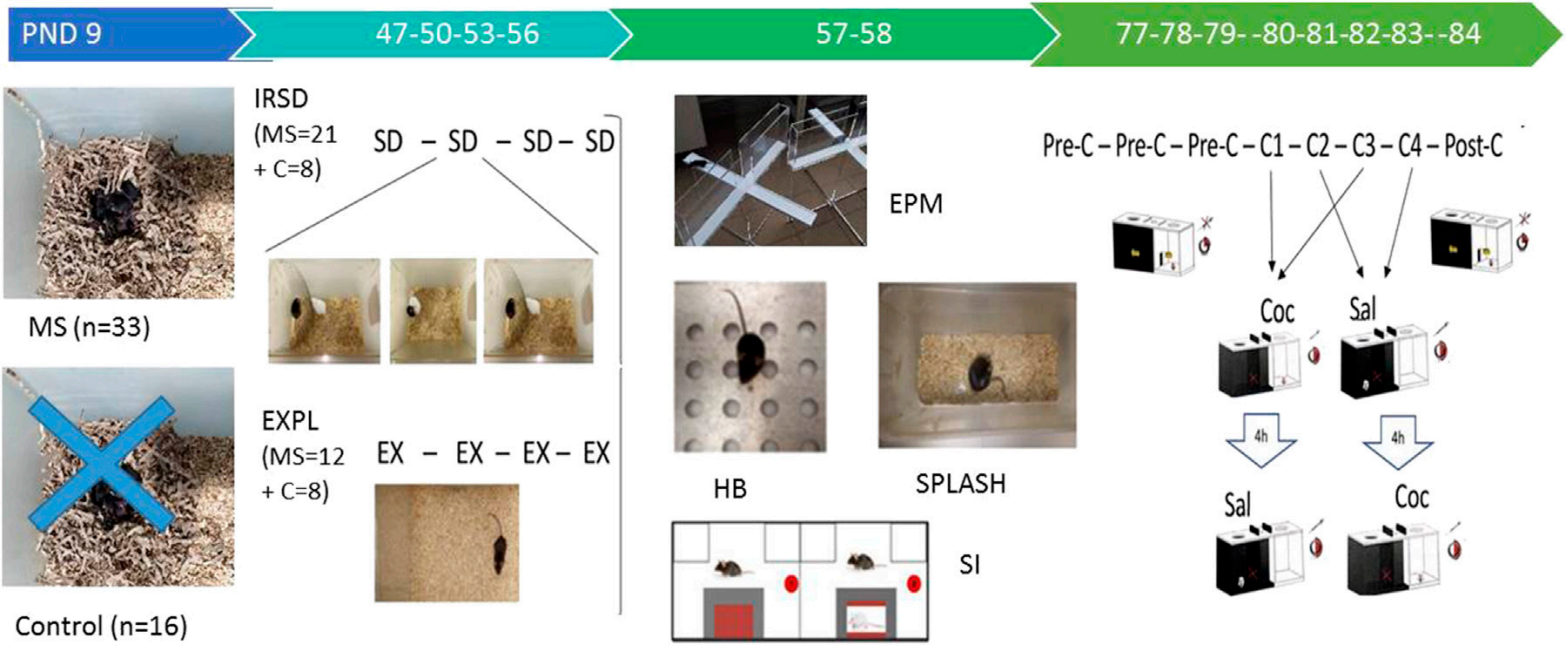
Timeline of experimental procedures. On postnatal day (PND) 9, a group of mice (n = 33) was exposed to a brief stress consisting of 6 h of maternal separation (MS), while a group of other mice did not undergo stress (control, *n* = 16). Subsequently, in late adolescence, mice exposed to MS and controls were divided into two groups in each case. The first MS group was exposed to intermittent repeated social defeat (group 1, MS + IRSD, *n* = 21). On PND 47, 50, 53 and 56, experimental mice were introduced into the cage of an aggressive opponent. Physical contact between them was allowed for only 5 min, during which the experimental mouse experienced social defeat (SD). On the same PND, the other MS group explored (EX) an empty cage (group 2, MS + EXPL, *n* = 12). Control mice without early life stress were divided into the same two groups (group 3, CONTROL + IRSD, *n* = 8; group 4, CONTROL + EXPL, *n* = 8). On PND 57, all mice performed the elevated plus maze (EPM), the hole board (HB) and the social interaction (SI) test. On PND 58, all mice performed the splash test. After an interval of 3 weeks, all mice underwent the conditioned place preference (CPP) paradigm. On PND 77, 78 and 79, mice underwent the pre-conditioning (Pre-C) phase. On PND 80, 81, 82, 83, mice performed four conditioning sessions (C1-C4), in which they received 1 mg/kg of cocaine (Coc) or saline (Sal) before being placed in the drug- or saline-paired compartment, respectively. On PND 84, mice underwent the post-conditioning (Post-C) phase.

The battery of behavioral tests took place on PND 57–58. On PND 57, the mice performed first the EPM, then the hole-board and then the social interaction test, with an interval of 1 h between each test. On PND 58 mice performed the splash test. Subsequently, after a 3-weeks interval, all the mice underwent the CPP procedure (see Figure 1). All experiments took place during the dark period (8.30–16.30 h), and mice were introduced into the dimly lit experimental room (different to that of the defeat and exploration procedures) 1 h prior to testing in order to facilitate adaptation.

### 2.4 Experimental Protocols

#### 2.4.1 Brief Maternal Separation

Repeated experiences of MS (PND2-12 or more, 3–8 h/day), often combined with early weaning (MSEW), constitute an animal model of early-life stress that reproduce the consequences of childhood adversity (George et al., 2010; Vetulani 2013; Bian et al., 2015) and allows researchers to evaluate their impact on the development of depression-like behaviour and on the response of animals to cocaine (Gracia-Rubio et al., 2016; Liu et al., 2018; Vannan et al., 2018; Castro-Zavala et al., 2020). Conversely, by enforcing MS for a short period, we aimed in this study to examine the impact of an acute episode of stress. Newborn mice (*n* = 54) were separated from their mothers for 6 h (9:00–15:00 h) on PND 9 (following a slight modification of the procedure employed in Llorente-Berzal et al., 2013). We selected PND nine for MS because this day marks the end of the neonatal period (PND3-9) and the initiation of postnatal transition (PND9-15) (Fox, 1965). In addition, mice show full retention 24 h after learning on PND 9 (Alleva and D’ Udine, 1987). During the separation, the mother was removed and placed in another cage (23 cm × 32 cm × 20 cm) with food and water access, while the pups remained in their home box. No specific procedure was used to keep the litter warm during this period, as the room temperature in the laboratory was maintained at 21°C and pups have a thick (almost complete) fur on PND 9, which helps thermoregulation. After 6 h, the mother was placed again with her litter. Weaning was carried out on PN21, during which the mice were separated by sex. Only male mice (*n* = 33) were used for the subsequent experiment. We randomly assigned each litter to the corresponding experimental group (5 litters in the SM + IRSD group, 3 litters in the SM + EXPL group, 2 litters in the CONTROL + IRSD group and 2 litters in the CONTROL + EXPL group). The 5 litters which comprised the 21 male mice of the SM + IRSD group were those in cages 1 (*n* = 4), 2 (*n* = 4), 4 (*n* = 5), 6 (*n* = 4) and 7 (*n* = 4). The 3 litters which comprised the 12 male mice of the SM + EXPL group were those in cages 3 (*n* = 3), 5 (*n* = 3) and 8 (*n* = 6). The 2 litters which comprised the eight male mice of the CONTROL + IRSD group were those in cages 9 (*n* = 4) and 10 (*n* = 4). The 2 litters which comprised the eight male mice of the CONTROL + EXPL group were those in cages 11 (*n* = 4) and 12 (*n* = 4).

#### 2.4.2 Repeated Social Defeat

On PND 47, 50, 53 and 56, the experimental mice (intruders) underwent the RSD procedure, which consisted of four agonistic encounters in which the animal was introduced into the home cage of a conspecific OF1 male mouse that had previously lived in isolation (aggressive opponent). Each encounter lasted for 25 min and consisted of three phases. During the first and last phases, it was protected from attack by a wire mesh wall, which allowed social threats from the aggressive resident (10 min). In the second phase, the wire mesh was removed and confrontation was allowed for 5 min, culminating in the defeat of the experimental mouse (for example, the adoption of an upright submissive position). For more details about the RSD procedure see Calpe-López et al. (2020). The non-defeated animals underwent the same protocol, but without the presence of an aggressive mouse, and simply explored (EXPL) the cage.

#### 2.4.3 Elevated Plus Maze

The effects of stress on anxiety-like behavior were evaluated on PND 57. The EPM consisted of two open and two enclosed arms (30 cm × 5 cm) and was elevated 45 cm above floor level. Mice have a natural aversion to open elevated areas; thus, anxiety is considered to be higher when open arms measurements (time spent and entries) are decreased (Rodgers and Johnson, 1995; Rodgers and Dalvi, 1997). The mice’s behaviour in the EPM was video recorded for 5 min and later analysed (Raton Time 1.0 software; Fixma SL, Valencia, Spain). The time and percentage of time [(open/open + closed) × 100] spent in the open arms, the number and percentage of open arm entries, time on the centre platform, total distance travelled, number of stretch-attend postures, number of head dips (protected or not) and rearing in the close arms were submitted to statistical analysis. For more details about the EPM apparatus and procedure see Calpe-López et al. (2020).

#### 2.4.4 Hole Board Test

The hole board test, used to evaluate novelty-seeking behavior, was carried out (PND 57) in a square box (28 cm × 28 cm × 20.5 cm) with 16 equidistant holes in the floor (Cibertec SA, Madrid, Spain) and equipped with photocells to detect the number of head-dips performed by the mouse during a 10-min period. For more details about the hole board test see Calpe-López et al. (2020).

#### 2.4.5 Social Interaction Test

On PND 57, the social behaviour of the mice was evaluated in an open field (37 cm × 37 cm × 30 cm), which contained a perforated plexiglass cage (10 cm × 6.5 cm × 30 cm). The mouse was allowed to explore the open field for 10 min on two occasions, separated for 2 min. On the first occasion (object phase), the plexiglass cage was empty. On the second occasion (social phase), a second mouse (OF1 strain) was put into the perforated cage and the experimental mouse was then reintroduced into the open field. In both phases, the time spent by the experimental mouse in the 8 cm area surrounding the perforated cage—considered the interaction zone (IZ) — was automatically registered (Ethovision 2.0, Noldus, Wageningen, Netherlands). An index of social interaction (ISI) was obtained [time spent in the IZ during the social phase/(time spent in the IZ during the social phase + time spent in the IZ during the object phase); Henriques-Alves and Queiroz, 2016]. It is common to use the ISI as the social preference-avoidance index (Krishnan et al., 2007). For more details about the social interaction test see Calpe-López et al. (2020).

#### 2.4.6 Splash Test

The splash test was carried out on PND 58. A 10% sucrose solution was sprayed on the dorsal coat of mice placed in a transparent cage (15 cm × 30 cm × 20 cm) containing bedding, which was designed to encourage grooming behaviour. Mice were recorded for 5 min, and the latency and frequency of grooming were analysed with the aid of a computerized method (Raton Time 1.0 software; Fixma SL, Valencia, Spain) by an observer who was unaware of the treatment administered. Lower frequency of grooming and higher latency to initiate this behaviour are considered to represent depressive-like behaviour (Smolinsky et al., 2009). For more details about the splash test see Calpe-López et al. (2020).

#### 2.4.7 Conditioned Place Preference

Three weeks after the last episode of social defeat, the mice underwent the CPP procedure (PND77-84). Eight identical Plexiglas boxes with two equal-sized compartments (30.7 cm long × 31.5 cm wide × 34.5 cm high) separated by a grey central area (13.8 cm long × 31.5 cm wide × 34.5 cm high) were employed. The compartments had different coloured walls (black vs. white) and contrasting floor textures (fine grid vs. wide grid). The position of the animals and their movement between compartments were detected by four infrared light beams in each of the compartments and six in the central area (MONPRE 2Z, Cibertec SA, Madrid, Spain).

The three phases of CPP took place during the dark cycle, and the assignment of the cocaine-paired compartment was carried out following a non-biased design (for more detail, see Maldonado et al., 2007). In summary, during pre-conditioning (Pre-C), the time spent by the animal in each compartment during a 15-min period was recorded, and those with a strong aversion or a preference for a particular compartment (less than 33% or more than 67% of the total time) were removed from the rest of study (*n* = 4). In the second phase (conditioning), lasting 4 days, experimental animals were administered saline and then confined to the vehicle-paired compartment for 30 min. Four hours later, they were injected with 1 mg/kg of cocaine and were immediately confined to the drug-paired compartment for 30 min. The sequence of the injections alternated each day (in the second and fourth conditioning session mice received cocaine first and then saline). During the third phase, or post-conditioning (Post-C), performed 24 h after the last conditioning session, the time spent by the untreated mouse in each compartment during a 15-min period was recorded. A conditioning score for each animal was calculated (time spent in Post-C minus time spent in Pre-C).

### 2.5 Statistical Analysis

The effects of MS and IRSD were evaluated using a two-way ANOVA with two between-subjects variables - Maternal Separation, with two levels (CONTROL and MS)—and Defeat, with two levels (EXPL and IRSD). Post hoc comparisons were performed with Bonferroni tests. The following behavioural measures were analysed: time, entries, percentage of time and percentage of entries in the open arms of the EPM, time on the centre platform of the EPM, total distance travelled in the EPM, number of stretch-attend postures, head dipping (protected or not) and rearing in the close arms of the EPM, number of dips in the hole board test, social interaction index (ISI), latency and frequency of grooming in the splash test, and conditioning score. In order to determine whether there was a relationship among the performances of mice in the different procedures, Pearson correlation tests were carried out. All statistical analyses were performed with the SPSS program.

## 3 Results

### 3.1 Effects of Maternal Separation in the EPM

**FIGURE 2:**
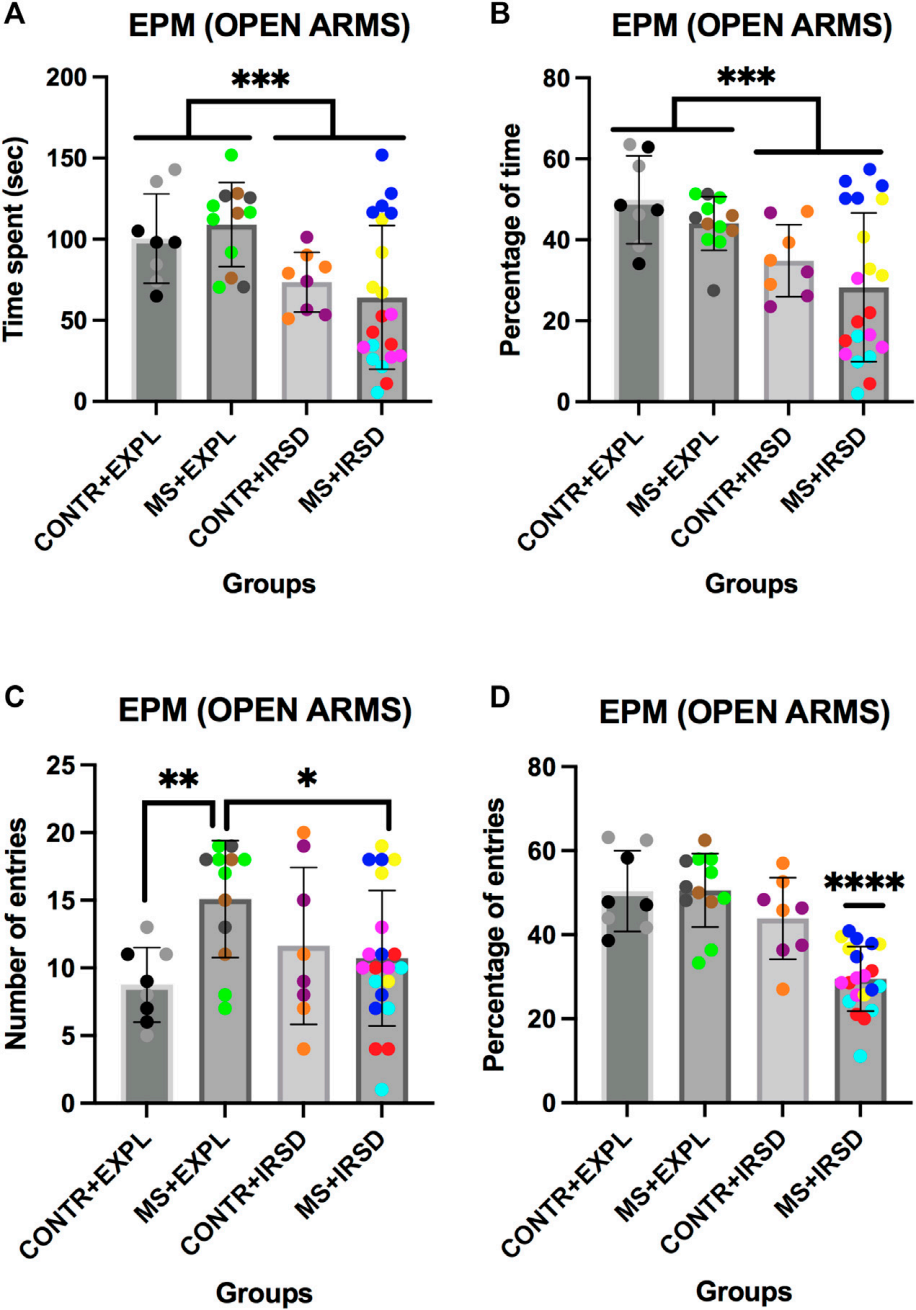
Effects of Maternal Separation (MS) and Intermittent Repeated Social Defeat (IRSD) on the Elevated Plus Maze (Open Arms Measurements). Control mice without early life stress explored an empty cage (CONTROL + EXPL, *n* = 8) or were exposed to SD (CONTROL + IRSD, *n* = 8) in the late adolescence (PND 47, 50, 53 and 56). Similarly, mice with early life stress (6 h of MS on PND9) explored an empty cage (MS + EXPL, *n* = 12) or were exposed to SD (MS + IRSD, *n* = 21) in late adolescence (PND 47, 50, 53 and 56). The animals’ behavior in the maze was evaluated on PND 57. **(A)** Bars represent the mean (±SD) time spent in the open arms (OA) of the maze for each group. ***p < 0.002, significant difference between mice exposed to IRSD and mice exposed to EXPL. **(B)** Bars represent the mean (±SD) percentage of time spent in the OA for each group. ***p < 0.002, significant difference between mice exposed to IRSD and mice exposed to EXPL. **(C)** Bars represent the mean (±SD) number of entries into the OA for each group. *p < 0.05, significant difference between the groups MS + EXPL and MS + IRSD; **p < 0.01, significant difference between the groups CONTROL + EXPL and MS + EXPL (post-hoc comparison of the Interaction Defeat X Separation). **(D)** Bars represent the mean (±SD) percentage of entries into the OA for each group. ****p < 0.001, significant difference of the group MS + IRSD with respect to the other groups (post-hoc comparison of the Interaction Defeat X Separation).

ANOVAs of the time (Figure 2A) and percentage of time (Figure 2B) spent in the open arms of the EPM revealed a significant effect of the variable Defeat {[F (1, 45) = 11.181, *p* < 0.002] and [F (1, 45) = 13.084, *p* < 0.001], respectively}, while the variable Separation and the Interaction Defeat X Separation were not significant. Mice exposed to IRSD (irrespective of whether they had been exposed or not to MS) spent less time and percentage of time in the open arms than mice exposed to Exploration.

ANOVAs of the number of entries in the open arms of the EPM (Figure 2C) revealed a significant effect of the Interaction Defeat X Separation [F (1, 45) = 6.540, *p* < 0.05], while the variables Defeat and Separation were not significant. Post-hoc analysis of the Interaction showed that mice exposed to MS (MS + EXPL group) performed a higher number of entries into the open arms in comparison to control mice (CONTROL + EXP) (*p* < 0.01); in addition, the group exposed to MS and defeat entered the open arms fewer times than mice only exposed to MS (MS + IRSD vs MS + EXPL, *p* < 0.05).

ANOVAs of the percentage of entries into the open arms of the EPM (Figure 2D) revealed a significant effect of the variables Defeat [F (1, 45) = 27.048, *p* < 0.001] and Separation [F (1, 45) = 7.170, *p* < 0.01], and of the interaction of the two [F (1, 45) = 7.527, *p* < 0.01]. Post-hoc analysis of the Interaction showed a lower percentage of entries into the open arms in the group exposed to MS and defeat (MS + IRSD) than mice exposed only to SM (MS + EXPL) or defeat (CONTROL + IRSD) (ps < 0.001).

**FIGURE 3:**
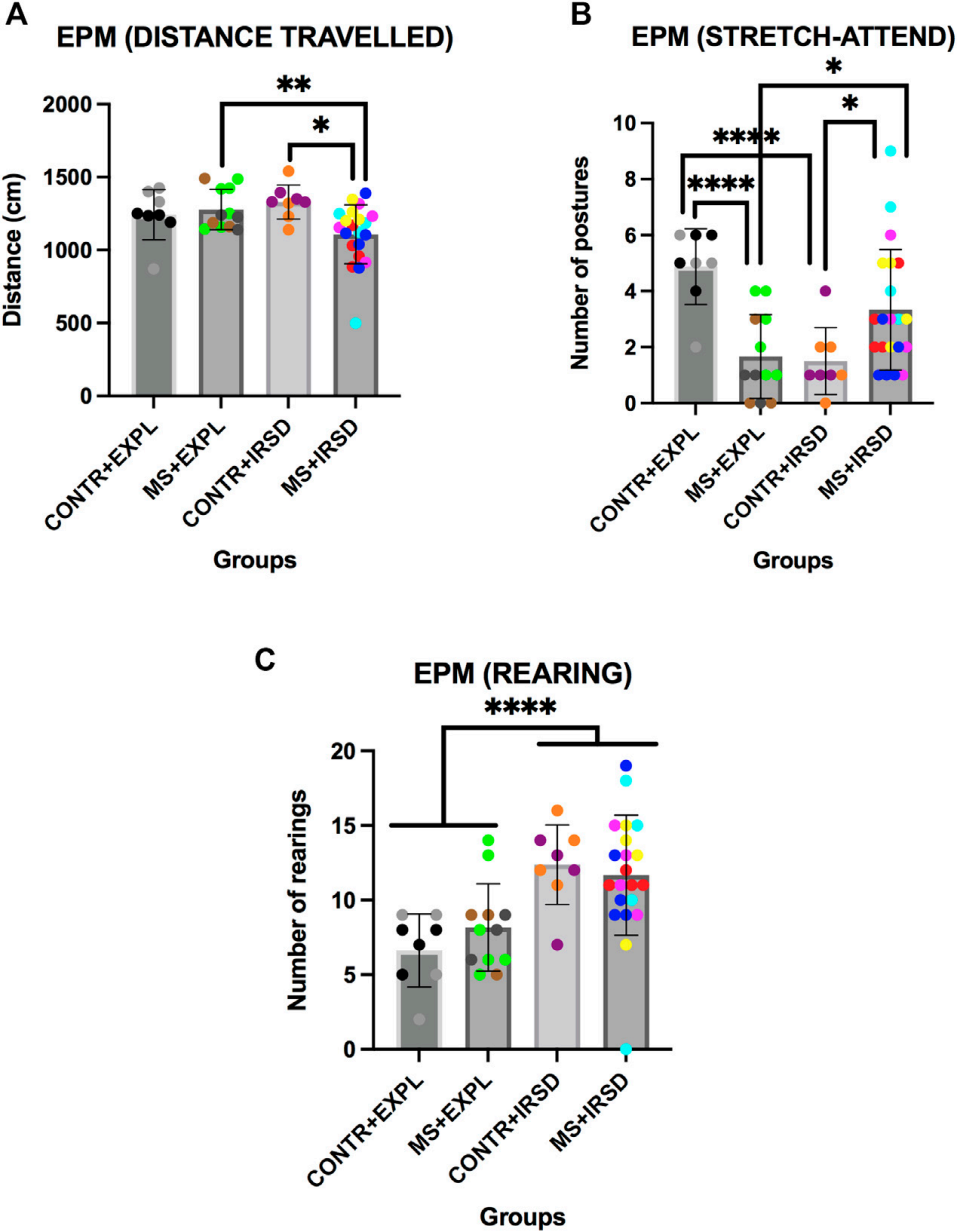
Effects of Maternal Separation (MS) and Intermittent Repeated Social Defeat (IRSD) on the Elevated Plus Maze (Additional Measurements). Control mice without early life stress explored an empty cage (CONTROL + EXPL, *n* = 8) or were exposed to SD (CONTROL + IRSD, *n* = 8) in the late adolescence (PND 47, 50, 53 and 56). Similarly, mice with early life stress (6 h of MS on PND9) explored an empty cage (MS + EXPL, *n* = 12) or were exposed to SD (MS + IRSD, *n* = 21) in late adolescence (PND 47, 50, 53 and 56). The animals’ behavior in the maze was evaluated on PND 57. **(A)** Bars represent the mean (±SD) distance travelled in the EPM for each group. *p < 0.05, significant difference between the groups CONTROL + IRSD and MS + IRSD; **p < 0.01, significant difference between the groups MS + IRSD and MS + EXPL (post-hoc comparison of the Interaction Defeat X Separation). **(B)** Bars represent the mean (±SD) number of stretch-attend postures in the EPM for each group. *p < 0.05, significant difference between the group MS + IRSD with respect to CONTROL + IRSD and MS + EXPL groups; ****p < 0.001, significant difference between the group CONTROL + EXPL with respect to CONTROL + IRSD and MS + EXPL groups (post-hoc comparison of the Interaction Defeat X Separation). **(C)** Bars represent the mean (±SD) number of rearing behaviors in the closed arms of the EPM for each group. ****p < 0.001, significant difference between mice exposed to IRSD and mice exposed to EXPL.

ANOVA of the total distance travelled in the EPM (Figure 3A) revealed significant effects of the Interaction Defeat x Separation [F (1, 45) = 21,415, *p* < 0.001]. Post-hoc comparison of the Interaction showed that the group SM + IRSD travelled a shorter distance than the SM + EXPL (*p* < 0.01) and CONTROL + IRSD (*p* < 0.05) groups.

ANOVA of the number of stretch-attend postures (Figure 3B) revealed significant effects of the Interaction Defeat x Separation [F (1, 45) = 21,415, *p* < 0.001]. Post-hoc comparison of the Interaction showed that the SM + IRSD group performed a greater number of stretch-attend postures than the CONTROL + IRSD and SM + EXPL groups (ps < 0.05). In addition, the CONTROL + EXPL group performed a greater number of stretch-attend postures than the SM + EXPL and CONTROL + IRSD groups (ps < 0.001).

ANOVA of the number of rearings in the close arms of the EPM (Figure 3C) revealed a significant effect of the variable Defeat [F (1, 45) = 5,858, *p* < 0.001]. A higher number of rearings was observed among mice exposed to defeat in comparison to those exposed to exploration.

ANOVAs of the time spent on the centre platform of the EPM and of head dipping (protected or not) did not reveal significant differences for the variables Defeat or Separation, or for their Interaction (data not shown).

### 3.2 Effects of Maternal Separation in the Social Interaction Test

**FIGURE 4:**
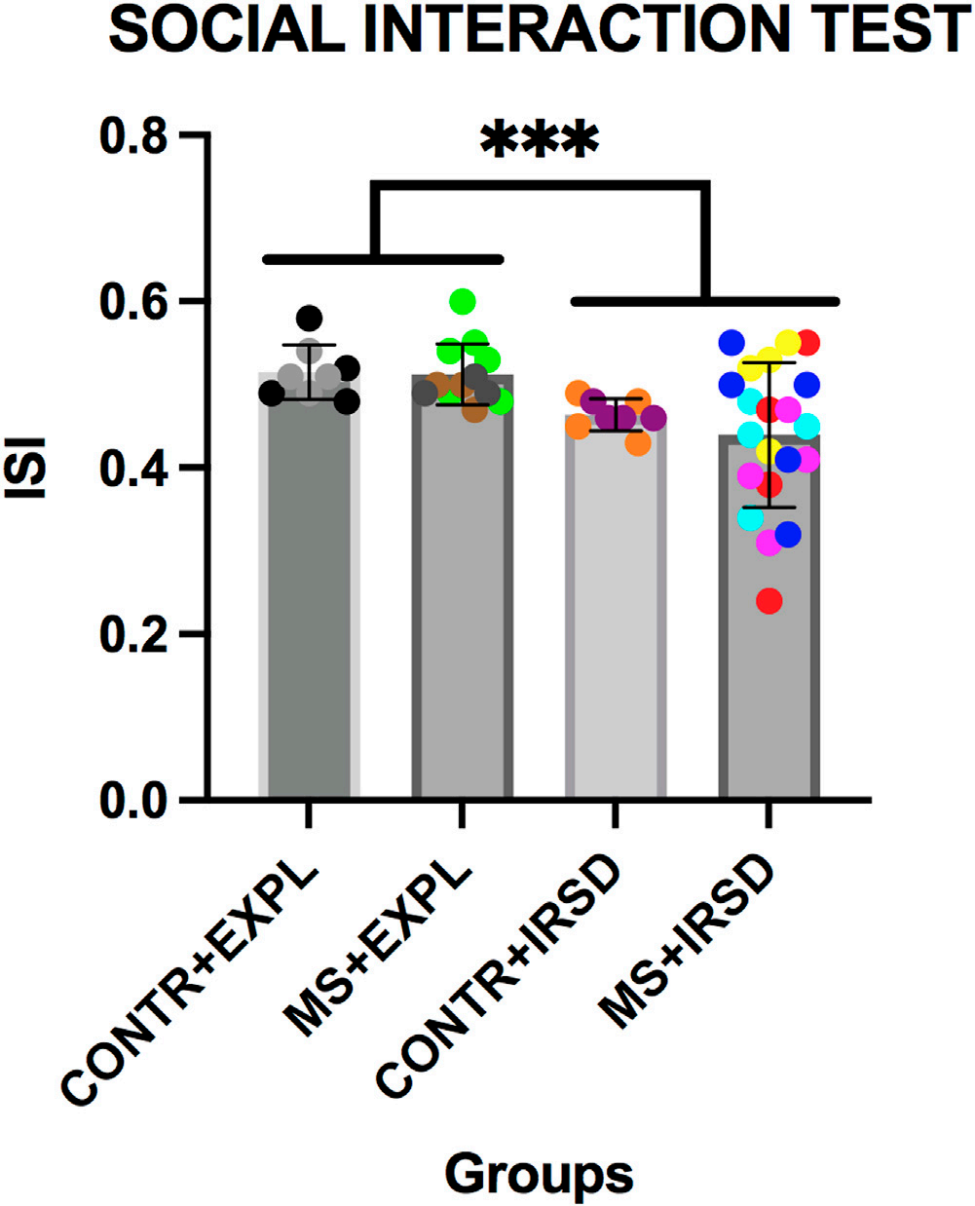
Effects of Maternal Separation (MS) and Intermittent Repeated Social Defeat (IRSD) on the Social Interaction Test. Control mice without early life stress explored an empty cage (CONTROL + EXPL, *n* = 8) or were exposed to SD (CONTROL + IRSD, *n* = 8) on the late adolescence (PND 47, 50, 53 and 56). Similarly, mice with early life stress (6 h of MS on PND9) explored an empty cage (MS + EXPL, *n* = 12) or were exposed to SD (MS + IRSD, n = 21) in late adolescence (PND 47, 50, 53 and 56). The behavior of mice in the social interaction test was evaluated on PND 57. Bars represent the mean (±SD) index of social interaction (ISI) in each group. ***p < 0.002, significant difference between mice exposed to IRSD and those exposed to EXPL.

ANOVA of data obtained in the social interaction test (Figure 4) revealed a significant effect of the variable Defeat [F (1, 45) = 10.476, *p* < 0.002], while the variable Separation and the Interaction Defeat X Separation were not significant. A lower ISI was observed among mice exposed to defeat in comparison to those exposed to exploration.

### 3.3 Effects of Maternal Separation in the Hole-Board Test

**FIGURE 5:**
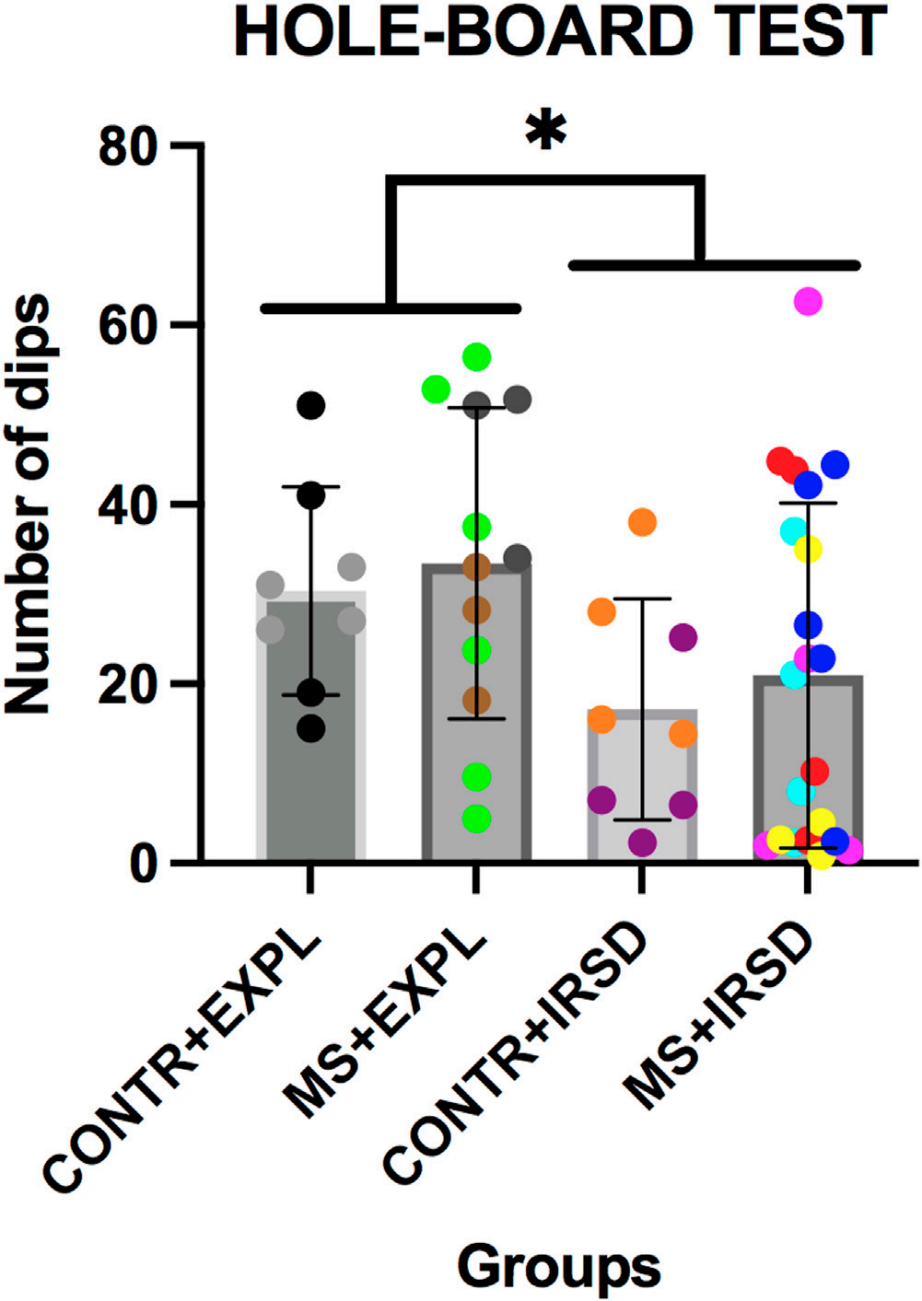
Effects of Maternal Separation (MS) and Intermittent Repeated Social Defeat (IRSD) on the Hole-Board Test. Control mice without early life stress explored an empty cage (CONTROL + EXPL, *n* = 8) or were exposed to SD (CONTROL + IRSD, *n* = 8) in late adolescence (PND 47, 50, 53 and 56). Similarly, mice with early life stress (6 h of MS on PND9) explored an empty cage (MS + EXPL, *n* = 12) or were exposed to SD (MS + IRSD, *n* = 21) in late adolescence (PND 47, 50, 53 and 56). The behaviour of mice in the hole board test was evaluated on PND 57. Bars represent the mean (±SD) number of dips in each group. *p < 0.05, significant difference between mice exposed to IRSD and mice exposed to EXPL.

ANOVA of the number of dips in the hole-board test (Figure 5) revealed a significant effect of the variable Defeat [F (1, 45) = 5.458, *p* < 0.05], while the variable Separation and the Interaction Defeat X Separation were not significant. A lower number of dips was observed among mice exposed to defeat in comparison to those exposed to exploration.

### 3.4 Effects of Maternal Separation in the Splash Test

**FIGURE 6:**
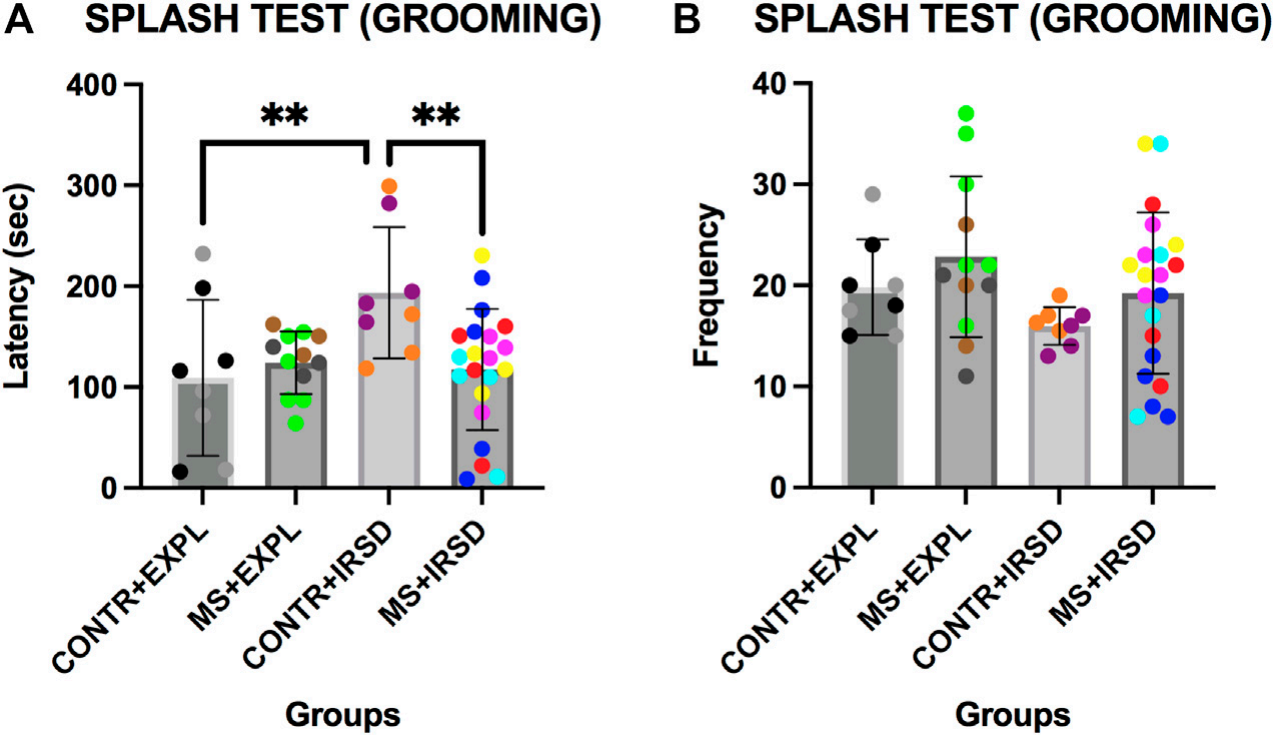
Effects of Maternal Separation (MS) and Intermittent Repeated Social Defeat (IRSD) on the Splash Test. Control mice without early life stress explored an empty cage (CONTROL + EXPL, *n* = 8) or were exposed to SD (CONTROL + IRSD, *n* = 8) in late adolescence (PND 47, 50, 53 and 56). Similarly, mice with early life stress (6 h of MS on PND9) explored an empty cage (MS + EXPL, n = 12) or were exposed to SD (MS + IRSD, n = 21) in late adolescence (PND 47, 50, 53 and 56). The behavior of mice in the splash test was evaluated on PND 58. **(A)** Bars represent the mean (±SD) latency of grooming behavior in each group. **p < 0.01, significant difference between the group CONTROL + IRSD with respect to CONTROL + EXPL and MS + IRSD groups (post-hoc comparison of the Interaction Defeat X Separation). **(B)** Bars represent the mean (±SD) frequency of grooming in each group.

ANOVA of the latency of grooming in the Splash Test (Figure 6A) revealed significant differences for the variables Defeat [F (1, 45) = 5.641, *p* < 0.05] and for the Interaction Defeat x Separation [F (1, 45) = 6.350, *p* < 0.05]. Post-hoc analysis of the Interaction showed that mice exposed only to defeat (CONTROL + IRSD) displayed a higher latency of grooming than non-defeated mice (CONTROL + EXPL) or mice exposed to both defeat and MS (MS + IRSD) (ps < 0.01). ANOVA of the frequency of grooming (Figure 6B) did not reveal significant differences for the variables Defeat or Separation or for their Interaction.

### 3.5 Effects of Maternal Separation in the CPP Paradigm

**FIGURE 7:**
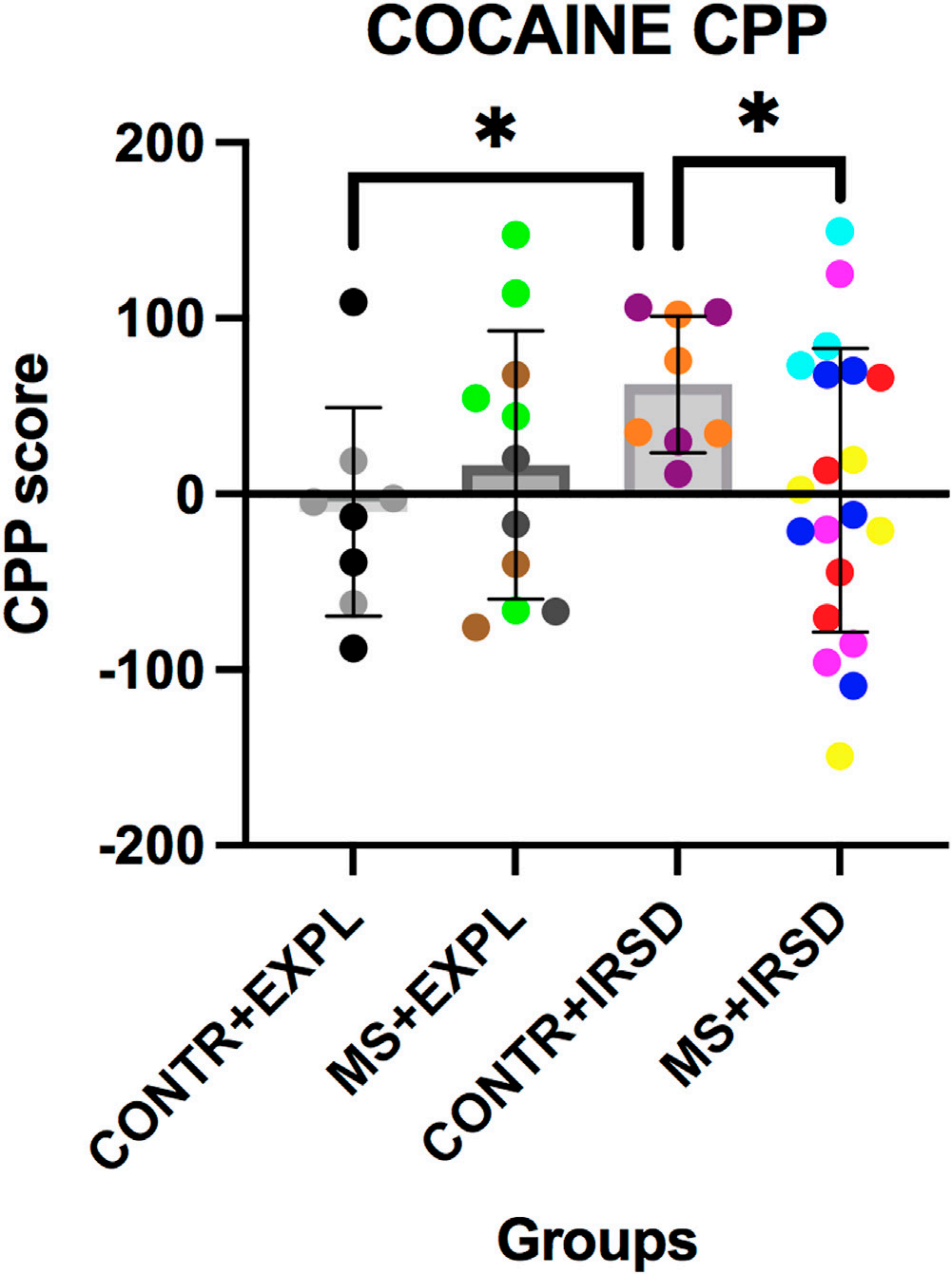
Effects of Maternal Separation (MS) and Intermittent Repeated Social Defeat (IRSD) on the Conditioned Place Preference (CPP) paradigm. Control mice without early life stress explored an empty cage (CONTROL + EXPL, *n* = 8) or were exposed to SD (CONTROL + IRSD, *n* = 8) in late adolescence (PND 47, 50, 53 and 56). Similarly, mice with early life stress (6 h of MS on PND9) explored an empty cage (MS + EXPL, *n* = 12) or were exposed to SD (MS + IRSD, *n* = 21) in late adolescence (PND 47, 50, 53 and 56). After behavioural tests on PND57-58 and an interval of 3 weeks, mice were conditioned with cocaine (1 mg/kg). Bars represent the mean (±SD) CPP score (in seconds) of each group. *p < 0.05, significant difference between the group CONTROL + IRSD with respect to CONTROL + EXPL and MS + IRSD groups (post-hoc comparison of the Interaction Defeat X Separation).

ANOVA of the conditioning scores (Figure 7) revealed a significant effect of the Interaction Defeat X Separation [F (1, 45) = 3.99, *p* < 0.05], while the variables Defeat and Separation were not significant. Post-hoc analysis of the Interaction showed that mice exposed only to defeat (CONTROL + RSD) had a higher conditioning score than non-defeated mice (CONTROL + EXPL) or mice exposed to both defeat and MS (MS + RSD) (ps < 0.05).

### 3.6 Correlations Between Behavioral Measures

Pearson tests revealed the existence of a significant positive correlation between the different measures of the open arms (time, percentage of time, entries and percentage of entries) (see Table 1). Some of these open arms measures also correlated with additional measurements evaluated in the EPM such as stretch-attend postures, protected and unprotected head dipping, and distance travelled in the EPM (see Table 1). In addition, the ISI positively correlated with the frequency of grooming (r = 0.297; *p* < 0.05), with time (r = 0.480; *p* < 0.001), percentage of time (r = 0.522; *p* < 0.001) and percentage of entries (r = 0.442; *p* < 0.001) in the open arms of the EPM, and with the distance travelled in the EPM (r = 0.352; *p* < 0.05).

**TABLE 1:** Correlations between measures of the elevated plus maze (EPM).

	TOA	EOA	PTOA	PEOA	DISTRAV	PROTHD	UNPROTHD	STR-ATT
TOA	---	0.416	0.934	0,63	0.101	0.086	0.402	-0.327
EOA	**	---	0.348	0.559	0.392	0.284	0.388	-0.284
PTOA	****	*	---	0.712	0.294	0.111	0.426	-0.238
PEOA	****	****	****	---	0.479	0.259	0.374	-0.249
DISTRAV	ns	**	*	***	---	0.268	0.471	-0,4
PROTHD	ns	*	ns	ns	ns	---	0.007	-0,57
UNPROTHD	**	**	**	**	***	ns	---	-0.178
STR-ATT	*	*	ns	ns	ns	ns	ns	---

Pearson correlations between different measures registered in the EPM. TOA, time spent in the open arms; EOA, number of entries in the open arms; PTOA, percentage of time in the open arms; PEOA, percentage of entries in the open arms; DISTRAV, distance travelled in the EPM; PROTHD, protected head dipping; UNPROTHD, unprotected head dipping; STR-ATT, stretch-attend postures. Upper section of the table: values of the Pearson correlation. Lower section of the table: level of statistically significant correlation between measurements (*p < 0.05, **p < 0.01, ***p < 0.001).

## 4 Discussion

The present study demonstrates that a brief MS prevents some effects of subsequent IRSD exposure in late adolescent mice, including increased latency of grooming behavior in the splash test and the potentiation of cocaine-induced CPP. However, MS did not modify the social avoidance and anxiety-like behavior induced by social defeat in our animals. Thus, we suggest that early stress induced in pups by a brief MS inoculates mainly against the long-term effects of subsequent social stress on vulnerability to cocaine reward.

In accordance with previous studies in our laboratory, we observed that mice exposed to IRSD during late adolescence displayed an increase in the rewarding effects of cocaine in adulthood, since defeated mice acquired CPP after being conditioned with a dose of cocaine, that is, known to be ineffective in inducing place conditioning in non-stressed mice (Montagud-Romero et al., 2017; García-Pardo et al., 2019; Calpe-López et al., 2020). On the other hand, as we expected, the MS procedure employed in our study did not alter the rewarding effects of cocaine. Conversely, other studies have demonstrated that exposure to MS increases the vulnerability of animals to cocaine reward (Matthews et al., 1999; Moffett et al., 2006; Viola et al., 2016; Alves et al., 2020). However, it is important to note that most of the studies in question used a combination of repeated episodes of MS (3 or 8 h per day, from PND2 to PND 12 or later) with early weaning (EW) on PND 14 or 17 (before PND 21, which is the natural moment for weaning) (Kikusui et al., 2004). In fact, MSEW is an animal model of early-life adversity (George et al., 2010; Vetulani 2013; Bian et al., 2015) and permits its impact on cocaine abuse to be evaluated (Liu et al., 2018; Vannan et al., 2018). MSEW causes an impairment of cocaine-induced behavioral sensitization, possibly due to a dysfunction of the dopaminergic system, a potential vulnerability factor for the development of substance use disorders (Gracia-Rubio et al., 2016). In addition, mice exposed to MSEW expressed higher cocaine intake, an enhanced vulnerability to the acquisition of cocaine self-administration, and an incapacity for this behavior to be extinguished (Castro-Zavala et al., 2020; Castro-Zavala et al., 2021a; Castro-Zavala et al., 2021b). In the CPP paradigm, MS (3h/day, from PND2 to PND14-15) also increased vulnerability to cocaine reward in adolescent rats (Alves et al., 2020) and mice (Viola et al., 2016), suggesting that this early life stress subsequently enhances the motivational salience of stimuli associated with cocaine.

The lack of an effect of our MS procedure on cocaine reward and the other behavioral parameters we have evaluated indicated that a single episode of MS (6 h, on PND9) is not a potent stressful event. In fact, our objective was to induce mild stress in early life in order to promote resilience to a subsequent stressful experience later in life, a phenomenon often referred to as stress inoculation (Ashokan et al., 2016). Indeed, the main contribution of our study is that is demonstrates how a brief MS can prevent the long-term effects of IRSD on the rewarding properties of cocaine. In particular, we observed that mice exposed to an episode of MS in early life and to repeated experiences of defeat in late adolescence behaved in the same way as non-stressed mice and did not acquire CPP after conditioning with a low dose (1 mg/kg) of cocaine. Thus, exposure to an episode of MS prevented enhancement of the sensitivity of mice to the rewarding effects of cocaine induced by IRSD. Although the effects of MS on the subsequent influence of stress on cocaine reward has not yet been evaluated, our results are in line with those of some studies which have demonstrated that neonatal stress procedures, including MS, can reduce the rewarding effects of drugs of abuse such as morphine (Boasen et al., 2009), MDMA (Llorente-Berzal et al., 2013) and cocaine (Hays et al., 2012). In addition, inoculation against stress early in life by means of disrupting dam-pup interactions (MS or limited bedding) was found to increase subsequent resilience to the effects of chronic SD stress on several physiological and behavioral parameters (Hsiao et al., 2016; Qin et al., 2019).

The protective effects of our MS procedure on the short-term effects of IRSD were less consistent. One or 2 days after the last episode of IRSD, late adolescent mice showed a reduction in all measurements related to the open arms of the EPM (considered to represent anxiety-like behavior; Campos et al., 2013), a reduced number of dips in the hole board (indicative of low novelty-seeking behaviour; Vidal-Infer et al., 2012), a deficit in social interaction, and a higher latency of grooming in the splash test (considered to represent depression-like behavior; Butelman et al., 2019). These results are in accordance with those of previous studies performed in our laboratory in which we observed that IRSD increased anxiety-like behavior in the EPM (García-Pardo et al., 2015; Calpe-López, et al., 2020), reduced social interaction (García-Pardo et al., 2015; Calpe-López, et al., 2020), induced social subordination (Rodríguez-Arias et al., 2016) and increased depression-like behavior (reduction in the frequency of grooming in the splash test) (Rodríguez-Arias et al., 2016; Calpe-López, et al., 2020). Conversely, our MS procedure did not alter the behavior of mice in any of the tests performed. These results contrast with data showing that MS increases social avoidance and induces anxiety- and depression-like behavior (Bian et al., 2015; Rana et al., 2015; Shin et al., 2016; Alves et al., 2020; He et al., 2020); nevertheless, it should be taken into account that these studies included repeated experiences of MS, while we used a single MS episode in order to induce a mild stress. In this line, a recent study has demonstrated that prolonged MS (3h/day, from PND1-21), but not short MS (15 min/day, from PND1-21), increases susceptibility to depression-like behavior when mice are exposed to chronic unpredictable mild stress in adulthood (Bian et al., 2021). In the present study, mice exposed to a brief MS became resilient to the depression-like behavior induced by exposure to IRSD in late adolescence (as indicated by changes in the latency of grooming). This result is especially important, as it underlines a close link between depression and cocaine abuse (Filip et al., 2013; Xu et al., 2020). In this sense, a recent study in our laboratory showed that resilience against the depression-like behavior (reduction in the frequency of grooming) observed a short time after IRSD is a behavioral trait related with subsequent resilience against the long-term effects of IRSD on cocaine reward (Calpe-López et al., 2020).

However, our MS protocol did not prevent other effects of IRSD, including anxiety-like behavior in the EPM, the reduced number of dips in the hole-board, and a deficit in social interaction. Some of these effects could also be related with the behavioral profile of mice that were resilient to the effects of IRSD on cocaine reward. In our previous study we observed that defeated mice that spent a lower percentage of time in the open arms of the EPM and performed a lower number of dips in the hole-board a short time after IRSD (animals exhibiting a greater concern for potential dangers in novel environments) are resilient against the potentiation of cocaine CPP induced by IRSD; conversely, defeated mice that displayed mild anxiety-like behavior and marked novelty-seeking behavior a short time after defeat were more vulnerable to the long-term effects of IRSD and developed CPP after conditioning with a low dose of cocaine (Calpe-López et al., 2020). In the present study, MS did not prevent the effects of IRSD in the EPM, since mice exposed to MS + IRSD exhibited a similar profile to mice exposed only to defeat (CONTROL + IRSD). Indeed, mice exposed to MS + IRSD showed a decrease in the percentage of entries in the open arms of the EPM and a reduction in the distance travelled in the EPM, neither of which were observed among mice exposed to IRSD or MS alone. On the other hand, mice exposed to MS + IRSD performed a greater number of stretch-attend postures than mice in the CONTROL + IRSD and MS + EXPL groups, but the MS + IRSD group was the only one that did not differ from non-stressed mice (CONTROL + EXPL group). Although an increase in stretch-attend postures has been interpreted as representing enhanced anxiety (Grewal et al., 1997), we observed higher values among mice in the CONTROL + EXPL group, which raises doubts about the true meaning of this measure. Stretch-attend postures in the EPM can be interpreted as a measure of risk behavior which occurs when the animal experiences an exploratory-anxiety conflict (Holly KS. et al., 2016). There was a positive correlation amongst all measurements in the open arms (time, entries, percentage of time, percentage of entries) and with unprotected head dipping, suggesting that this latter measure is also indicative of lower anxiety. Similarly, the distance travelled correlated positively with number of entries, percentage of entries and percentage of time in the open arms. All these measurements were higher in non-stressed mice than in defeated animals, thus indicating anxiety-like behavior irrespective of whether or not there was exposure to MS. Conversely, there was a negative correlation between the number of stretch-attend postures and the number of entries and time spent in the open arms, but not with the percentages of these measures. These results may simply indicate that mice perform stretch-attend postures more frequently when they are in closed arms than when they are in open arms.

The fact that MS did not ameliorate the social interaction deficit induced by IRSD also contrasts with our previous study in which resilient defeated mice that did not develop cocaine CPP were also characterized by a lack of social avoidance (Calpe-López et al., 2020). From our point of view, the most plausible explanation is that a more pronounced early-life stress is necessary to induce inoculation against the short-term effects of IRSD on the EPM and social interaction test. In support of this hypothesis, it has been observed that repeated MS (1 h/day, from PND 3–21) alleviates the increased anxiety-like behavior induced by chronic SD stress in adulthood (Qin et al., 2019). Similarly, fragmented dam-pup interactions during PND2-9 (by limiting bedding and nesting material in the cage) was seen to reduce the social interaction deficit induced by chronic SD stress (Hsiao et al., 2016). While MSEW induced a depression phenotype and increased cocaine abuse (Liu et al., 2018; Vannan et al., 2018), we observed that a mild MS stress prevented the depression-like behavior and potentiation of cocaine reward induced by IRSD, though not enough to counteract other effects of IRSD. Future studies need to determine the level of MS that induces positive effects and effectively reverses all the effects of subsequent stress exposure. Age and sex could be mediating factors in the inoculation against stress by MS, since adolescence is a period of extreme vulnerability to the effects of drugs of abuse and the development of mental disorders (Dow-Edwards et al., 2019), and there are distinctive sex differences in these disorders, including substance use disorders (Becker, 2016; Li et al., 2017). Thus, it could be relevant to evaluate the effects of MS in female mice exposed to vicarious social defeat stress. Furthermore, the genetic predisposition of subjects to low or high emotional reactivity may be an important factor in determining the positive or negative effects of MS. Rats with a high novelty response and low anxiety/depression levels have been found to be resilient to the negative physiological effects of MS stress (3 h/day, from PND1-14) (Clinton et al., 2014). The same protocol of MS induced social avoidance and anxiety-, and depressive-like behaviors in Wistar rats, but had the opposite effects in Wistar-Kyoto rats, an animal model of comorbid depression and anxiety (Rana et al., 2015). The present study has other limitations. First, we have evaluated only the effects of MS on the CPP induced by a low dose of cocaine. This single-dose experiment provided limited information, and so a complete dose-response study would need to be performed in order to draw solid conclusions about the effects of MS on cocaine reward. Second, the design of our study can induce litter effects; in other words, mice from the same litter are phenotypically more similar than mice from different litters. Litter effects account for an elevated percentage of variability and can mask the true effects of an experimental treatment. Thus, the impact of litter-to-litter variability should be controlled and minimized in order to enhance the rigor and reproducibility of the results observed in this study.

Our results suggest that inoculation against stress early in life through a brief episode of MS increases subsequent resilience to some of the negative effects of IRSD stress, since it prevents the development of depression-like behavior in mice defeated in late adolescence and long-term enhancement of their sensitivity to cocaine reward in adulthood. In terms of the mechanisms underlying such adaptive changes, we hypothesize that the glutamatergic system and the hypothalamus pituitary adrenal (HPA) axis are involved. A moderate MS that prevented the increase in anxiety-like behavior induced by chronic SD stress in adulthood was also seen to prevent the hyperactivity of glutamatergic transmission in the basolateral amygdala induced by this kind of stress (Qin et al., 2019). In addition, mice exposed to moderate early life stress show less social interaction deficits after chronic SD stress (Hsiao et al., 2016), and exhibit a significant decrease in the corticosterone response to a subsequent stressful event (Plotsky and Meaney, 1993; Hsiao et al., 2016). No studies have been performed about the inoculating effect of MS on the subsequent response of stressed animals to drugs of abuse. However, studies of the mechanisms than underlie the effects of more stressful protocols of MS on the rewarding properties of cocaine have revealed that MS modifies the activity of AMPA and NMDA receptors in structures of the brain reward circuit and other areas involved in the learning of cocaine-cue association (Ganguly et al., 2019; Castro-Zavala et al., 2020; Castro-Zavala et al., 2021b). MSEW was seen to enhance glutamatergic function in the nucleus accumbens and increase excitability of ventral tegmental area DA neurons (Castro-Zavala, et al., 2021a). Moreover, the impairment in reward function induced by MS was reversed by blocking glutamate signaling during adolescence (O'Connor et al., 2015). Additionally, a link between MS and cocaine reward and levels of tumor necrosis factor (Ganguly et al., 2019), brain-derived neurotrophic factor (BDNF, Viola et al., 2016) or TrkB receptors (Orso et al., 2017) has been demonstrated. We hypothesize that the protective effects of a brief MS on the subsequent potentiation of cocaine reward induced by IRSD is also mediated by the glutamatergic system and modifications of both the HPA system and different signaling pathways. In previous studies in our laboratory, we observed that social defeat decreased the expression of several subunits of NMDA and AMPA receptors (García-Pardo et al., 2019), and that the antagonism of NMDA receptors before each episode of defeat prevented the potentiation of cocaine CPP induced by IRSD. The same effect has been observed with the antagonism of CRF receptors (Ferrer-Pérez et al., 2018). Dopaminergic pathways, BDNF signaling and TrkB receptors also play a role in the effects of IRSD on cocaine reward (Montagud-Romero et al., 2017). Future research should attempt to unravel the cellular and molecular mechanisms underlying the protective effect of brief or moderate protocols of MS on the negative consequences of social defeat stress. In addition, the mother’s reactions when returned to her litter may contribute to the inoculation against stress induced by brief MS. The potential impact of mother/pup interactions before and after MS on the outcomes observed in the offspring should be evaluated by future research. Such studies could help to develop new intervention approaches for the prevention of stress-related disorders and new therapeutic strategies to treat vulnerable individuals at risk of developing a drug use disorder following stressful experiences.

## Data Availability

The raw data supporting the conclusion of this article will be made available by the authors, without undue reservation.
